# Differentially pumped spray deposition as a rapid screening tool for organic and perovskite solar cells

**DOI:** 10.1038/srep20357

**Published:** 2016-02-08

**Authors:** Yen-Sook Jung, Kyeongil Hwang, Fiona H. Scholes, Scott E. Watkins, Dong-Yu Kim, Doojin Vak

**Affiliations:** 1 CSIRO Manufacturing Flagship, Bag 10, Clayton South, Victoria 3168, Australia; 2 School of Materials Science and Engineering, Gwangju Institute of Science and Technology, Gwangju 500-712, Republic of Korea

## Abstract

We report a spray deposition technique as a screening tool for solution processed solar cells. A dual-feed spray nozzle is introduced to deposit donor and acceptor materials separately and to form blended films on substrates *in situ*. Using a differential pump system with a motorised spray nozzle, the effect of film thickness, solution flow rates and the blend ratio of donor and acceptor materials on device performance can be found in a single experiment. Using this method, polymer solar cells based on poly(3-hexylthiophene) (P3HT):(6,6)-phenyl C61 butyric acid methyl ester (PC_61_BM) are fabricated with numerous combinations of thicknesses and blend ratios. Results obtained from this technique show that the optimum ratio of materials is consistent with previously reported values confirming this technique is a very useful and effective screening method. This high throughput screening method is also used in a single-feed configuration. In the single-feed mode, methylammonium iodide solution is deposited on lead iodide films to create a photoactive layer of perovskite solar cells. Devices featuring a perovskite layer fabricated by this spray process demonstrated a power conversion efficiencies of up to 7.9%.

Solution processed solar cells are a promising renewable energy technology. The technology has received a great deal of attention because it benefits from a cost-competitive manufacturing process and can produce solar cells with different form factors than conventional silicon solar cells. Given the significant potential of this technology, intensive research in this area has been carried out in the past decade. As a result, the power conversion efficiency (PCE) of solution processed solar cells has increased rapidly^1^,^2^,^3^. In particular, one class of solution processed solar cell, the organic-inorganic hybrid perovskite solar cells (PeSCs) have shown dramatic progress with certified PCEs of up to 20.1% being reported^4^. Polymer solar cells (PSCs), another type of solution processed solar cell, have also shown a rapid increase in record efficiency with PCEs of more than 10% being reported^5^. Although the efficiencies of PeSCs now exceed those of PSCs, PSCs are still important as a more developed technology. In particular, large scale manufacturing of PSCs has been demonstrated^6^,^7^,^8^ while PeSC development is still at a very early stage^9^.

Progress in PSCs has been achieved through the development of new materials followed by process optimisation. Once a new material is developed, the optimal processing conditions of the new material are experimentally determined. Therefore, various processing parameters need to be tested in a wide range of combinations. If a good combination of processing parameters is found, devices will demonstrate promising performance. In this case, the process can be further optimised to achieve the maximum efficiency possible given the material properties. There are many processing parameters that must be considered such as the donor/acceptor ratio, solvent, concentration, coating conditions, temperature, additive type and the concentration of additives^10^,^11^,^12^,^13^,^14^. Large variations in the performance of a polymer as a result of different processing conditions have been reported^15^,^16^,^17^. As numerous combinations of the processing parameters are possible, it is difficult to test all the possibilities in a typical research and development process unless promising results are obtained at the screening stage. As a result, some materials that have unusual optimal processing conditions may be overlooked despite having great potential. Spin coating is the most common process for fabricating PSCs in the laboratory and has been also used for material screening^18^. The process is very useful for producing thin, uniform films on small rigid substrates. Therefore, high performance devices requiring a uniform film with an ideal film thickness can be easily fabricated. However, the process is not ideal for screening various processing conditions due to the large material consumption the process entails. Most of the polymer material is wasted during the spinning process. In addition, only one combination of processing parameters can be tested per sample.

Accordingly, a more efficient screening method has been highly sought after. Accelerated bar coating^19^ has been reported as one possible screening method whereby wedge-shaped photoactive layers of varied thickness were fabricated. The effects of film thickness on device performance could therefore be efficiently tested in a single experiment. A dual-feed slot-die coater with a differential pumping system has also been reported as a high throughput screening tool^20^. The donor/acceptor ratio was changed during the roll-to-roll coating process by the differential pump and various donor-acceptor ratios were tested in a single, continuous coating process enabling the effects of film thickness to be tested using a single-feed mode. Spray deposition is another method for the high throughput screening of materials. Uniquely, the process consists of numerous depositions of small droplets instead of forming wet film all at once. This process enables controlled deposition such that various parameters can be used within a small area through the use of position control. It also allows finely controlled film deposition by using *in situ* thickness monitoring during the deposition^21^.

In this study, we report a spray deposition method as a high throughput material screening tool for PSCs and PeSCs. Since our first demonstration of spray coated PSCs^22^, it has been widely used for the fabrication of PSCs due to the versatility and scalability of the process^23^. Although the process typically produce much more rough films compared to spin coated ones^24^, high performance PSCs^25^,^26^ as well as PeSCs^27^ have been demonstrated. The mixing of materials during the deposition process makes this technique a very useful screening tool^28^. Various deposition parameters can be tested in one deposition process by introducing *in situ* mixing and position control. To realize high throughput screening, a computer controlled spray deposition system with a dual-feed nozzle has been developed for the deposition of photoactive materials with precise control of deposition parameters including solution flow rate, blend ratio and coating speed. Two syringe pumps are used to control solution flow rates independently during the deposition process. Various compositions can be formed on substrates via *in situ* blending. The system can also be used in single-feed mode to test a single parameter. Therefore, we used both single and dual-feed modes to screen deposition parameters for the fabrication of photoactive layers in this study.

## Results and Discussion

**Figure 1:**
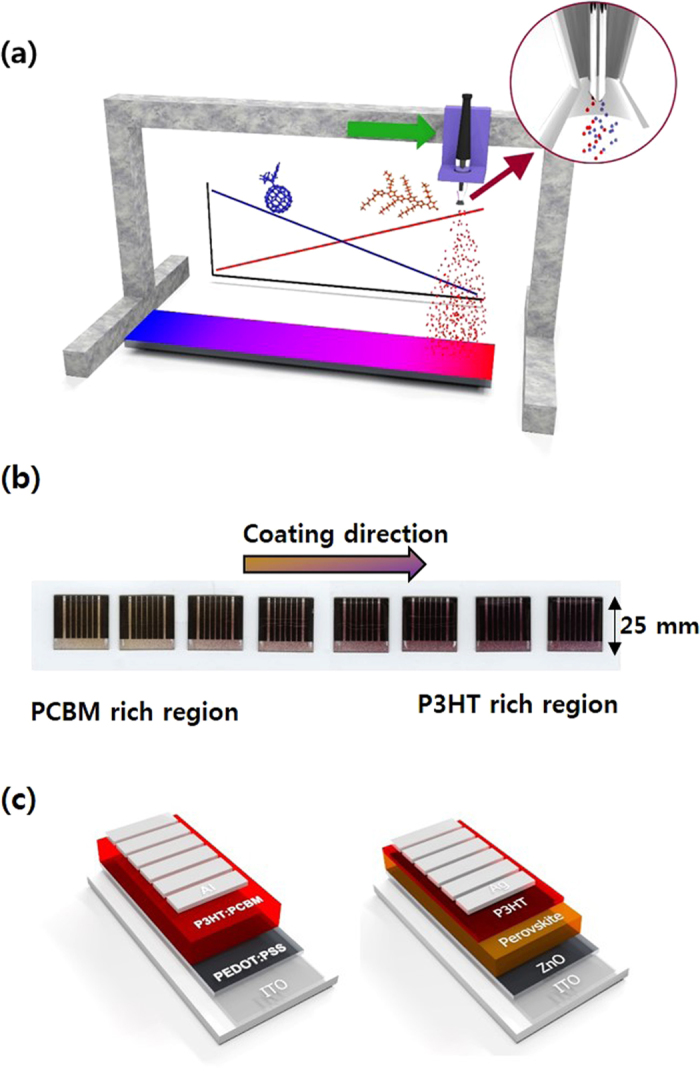
(**a**) Schematic illustration of a dual-feed spray deposition system for rapid formulation screening. The system is also used in single-feed mode with a differential pumping system for the screening of deposition parameters. (**b**) Scanned image of devices fabricated from a single deposition process using dual-feed spray. (**c**) Device configurations used in this study.

A schematic illustration of the dual-feed spray deposition process used in this study is shown in Fig. 1(a). A commercial air-brush was modified to feed two solutions separately by introducing two long syringe needles in place of the original needle. The syringe needles have bevelled tips that face in opposite directions so that atomisation can occur separately. From our previous experiences, we found soft tubes with a large internal diameter causes problems such as large dead volume and, more importantly, volume expansion when high pressure was applied. To overcome these problems, we used 1/16″ hard tube (PEEK) with a 0.02″ internal diameter, which was designed for HPLC. Therefore, dead volume was minimized (~0.1 ml for 50 cm length tube) and deposition could be start/stop almost immediately after syringe pump start/stop. The spray nozzle slowly moves at constant speed from one end of the sample to the other while gradually changing flow rates. As the solution flow rate of one material decreases, the flow rate of the other material increases to maintain a constant level of total solution. The effect of the blend ratio of the donor and acceptor materials at approximately the same thickness can be determined from this experiment. The system can also be used in single feed-mode. In this mode, only one solution is fed through the nozzle with a gradually increasing solution flow rate. Figure 1(b) shows an example of dual-feed spray coated devices. Coating was started from left side with high flow of PCBM solution and low flow of P3HT solution. Spray head was moved to right direction and proportion of P3HT was increased while keeping constant total solution flow. Gradual colour change clearly show controlled composition of two materials. We applied this process for both PSCs and PeSCs. Device configurations used in this paper are shown in Fig. 1(c).

**Figure 2:**
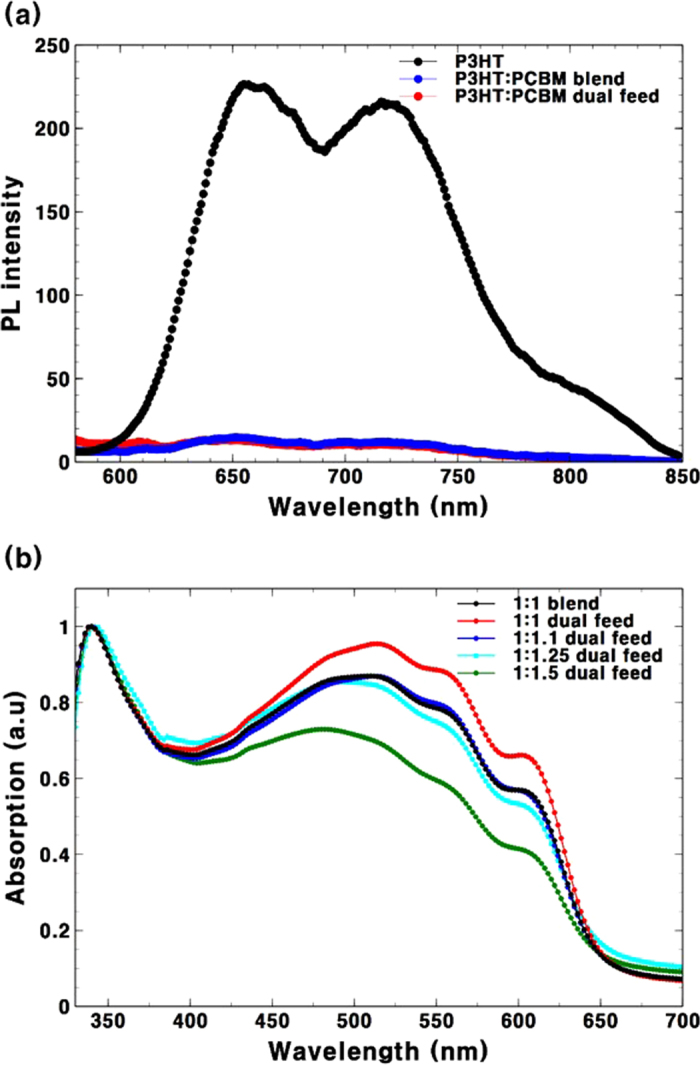
(**a**) PL spectra of P3HT and P3HT:PC_61_BM blends from a pre-mixed solution and *in situ* blending by dual-feed spray deposition. (**b**) Absorption spectrum of *in situ* blended P3HT:PC_61_BM films fabricated by dual-feed spray deposition normalised based on the peak of PC60BM at 340 nm. The spectrum of a 1:1 blend obtained from a pre-mixed solution is also shown as a reference.

As the first application, we applied the screening process to the well-known P3HT:PC_61_BM combination as this blend is the most-studied system in polymer solar cell research^29^. Results obtained from the experiments could then be easily compared with results from previous studies. Before the screening process, the quality of the *in situ* blended film was tested by a simple photoluminescence (PL) quenching experiment. To be a photoactive layer in bulk heterojunction solar cells, the donor and acceptor materials should be well mixed and have nano-scale phase separation to achieve efficient charge dissociation at the interface of the donor and accepter domains. PL quenching has been widely used to test the degree of phase separation^30^,^31^. Therefore, we prepared P3HT:PC_61_BM films from both a pre-mixed blend solution and separated P3HT and PC_61_BM solutions. Separated P3HT and PC_61_BM solutions were blended *in situ* on a glass substrate by a dual-feed spray deposition. PL spectra from the blended films and from a pure P3HT film are shown for reference in Fig. 2(a). The *in situ* blended film shows the same degree of quenching as the film formed from the pre-mixed solution. This result confirms that the donor and acceptor materials were well mixed and there is no significant proportion of large P3HT domains in the film. After confirming the quality of the blended film, the composition of P3HT and PC_61_BM in the blended films was calibrated. In the spray deposition process, solutions atomised into femtolitre-size droplets^32^. Most droplets land on the substrate but some dry in the air and turn to dust. We observed this dust on all surfaces of the spray box. Therefore, the deposition yield^33^ of the solutions needs to be considered. The composition of the blended film was calibrated by comparing the absorbance spectra of the blended films as shown in Fig. 2(b). *In situ* blended films were prepared from P3HT and PC_61_BM solutions in chlorobenzene. Controlling the solution feed ratio allowed the formation of a different composition. Blended films were also prepared from a pre-mixed P3HT:PC_61_BM (1:1) blend solution as a reference. To see the relative content of P3HT, the spectrum was normalised based on the peak of PC_61_BM at 340 nm. Figure 2(b) clearly shows the different deposition yields of the polymer and PC_61_BM. The absorption spectrum from the pre-mixed solution with a 1:1 ratio closely matched the *in situ* blended film with a 1:1.1 ratio of P3HT and PC_61_BM. This implies that the transfer yield of P3HT is a little higher than that of PC_61_BM under similar deposition conditions. Based on this calibration result, the solution flow rate of the PC_61_BM was multiplied by 1.1 in all experiments in this work.

**Figure 3:**
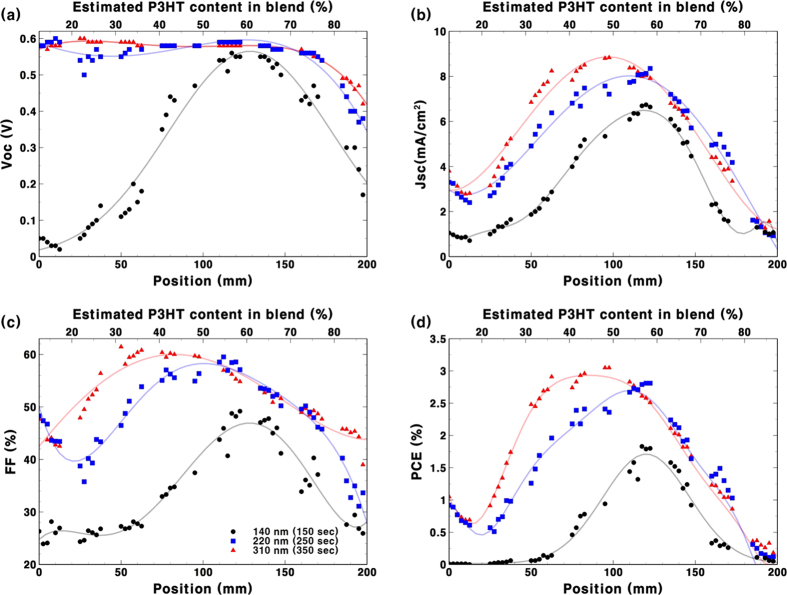
(**a**) V_oc_ (**b**) J_sc_ (**c**) FF and (**d**) PCE of P3HT:PC_61_BM based devices fabricated by dual-feed deposition of P3HT and PC_61_BM solutions. The solution flow rate of the P3HT is ramped up while the spray nozzle moves from position zero to 200 mm while the sum of the P3HT and the PC_61_BM solutions is kept constant. Film thickness is controlled by the total deposition time over the 200 mm coating length and is measured at the mid-point.

To fabricate devices with various compositions using the dual-feed spray deposition method, a differential pumping system was used together with a motorised spray nozzle. Films could be fabricated on a long substrate to be a large single cell and the current output obtained by a photo current mapping technique as used with the accelerated bar coating method^19^. The drawback of this method is that other device parameters cannot be tested. Therefore, we used our typical device configuration for multiple small pixels on a substrate. Eight PEDOT:PSS coated Indium tin oxide (ITO) substrates with dimensions of 25 mm × 25 mm were placed under the spray coater to produce a graded composition over a 200 mm length. Each substrate consisted of 6 cells with a 2.5 mm interval in the middle of the substrate which causes the gap between the devices, as shown in Fig. 1(b). A total of 48 different devices could be fabricated from a single deposition process. Three depositions were carried out with different total deposition times while keeping the deposition profile the same in order to produce different thicknesses. Performance characteristics of the spray-coated device can be seen in Fig. 3. Because the composition of the blend was changed as the spray head moved, a change in position equates to a change in composition. Thus, at position zero, the blend is almost pure PC_61_BM and at a position of 200 mm the blend is almost pure P3HT. Ratios of the two materials between zero and 200 mm are shown at the top of Fig. 3. However, the amount of material is only estimated from the input. Before analysing the performance characteristics, we found an unexpectedly high efficiency at the starting point (PC_61_BM rich region). We attribute this to an artefact of the deposition system. The deposition process starts with filling the nozzles with two solutions and moving them to the home position. Excess ink is deposited to the waste bin as the head moves to the first substrate at a fixed solution flow rate (0.01 ml/min for P3HT and 0.077 ml/min for PC_61_BM). The nozzle then slowly moves over the substrate with gradually changing solution flow rates. However, at the starting point, some residual P3HT solution in the needle can escape and result in an excess amount of P3HT on the film. As there is only a very small amount of P3HT at the starting point, a very small increase in this solution at this position could result in a significant change in the device performance. This intermittent problem was unavoidable but it only affected the sample area outside the region of interest. Other than this problematic region, clear performance trends can be seen in the Fig. 3. The block dots show devices with a film thickness of 140 nm (as measured at the device mid-point) with a 1:1 material ratio. In PCBM rich region, devices showed low V_oc_ caused by low coverage of the layer as previously shown in very thin spray coated devices^21^,^22^. Although the problem was observed in thinner layers in case of P3HT rich formulation, the problem could be more serious with the non-polymeric PCBM rich region. The batch shows a clear trend up to 1.8% PCE with a material ratio of P3HT:PC_61_BM ~6:4. With increased thickness (corresponding to a longer total deposition time), devices showed improved performance. At the same time, the optimum performance point was shifted to a more PC_61_BM rich region. The thickest device, with a 310 nm photoactive layer, showed a PCE of up to 3.0% with a 1:1 ratio. This trend is consistent with the fully optimised conditions reported by many research groups^34^. Devices with a P3HT rich blend typically show high performance when the active layer is around 100 nm thick and devices with a 1:1 blend typically show high performance when the active layer is over 200 nm thick. The result demonstrates that the reported screening process is a useful method to find a good starting point for the further optimisation of new materials.

We expanded the application of the deposition system to PeSCs. As previously discussed, PeSCs are an emerging technology with rapidly improving device performances. However, there are still many challenges for commercialisation. One of challenges will be finding a new formulation with improved properties. CH_3_NH_3_PbI_3_ and CH_3_NH_3_PbI_3−x_Cl_x_ have been most widely used and although these materials have shown promising optical and electronic properties, instability in the presence of moisture and the intrinsic toxicity of lead compounds have been issues^35^,^36^,^37^. The high throughput screening method reported here would be very useful if it can be used for new perovskite materials. To demonstrate this capability, we fabricated partially spray processed PeSCs. There are two approaches to this fabrication process. The first approach is a single step deposition process and the second approach is a sequential deposition process. The former approach uses a blended solution of a lead halide compound and an organic compound and the latter approach requires the fabrication of a lead halide film followed by a conversion process to perovskite. Although the former process is simpler and seems ideal for production, it has been reported that the formation of defect-free films is very challenging due to the rapid crystallisation of perovskite^38^,^39^. Therefore, a sequential deposition process was developed and has been widely used^40^. We also found that it was very challenging to form defect-free perovskite films by a one-step spray coating process. Therefore, we adopted the more reliable sequential process to test the spray deposition method for the fabrication of PeSCs.

**Figure 4:**
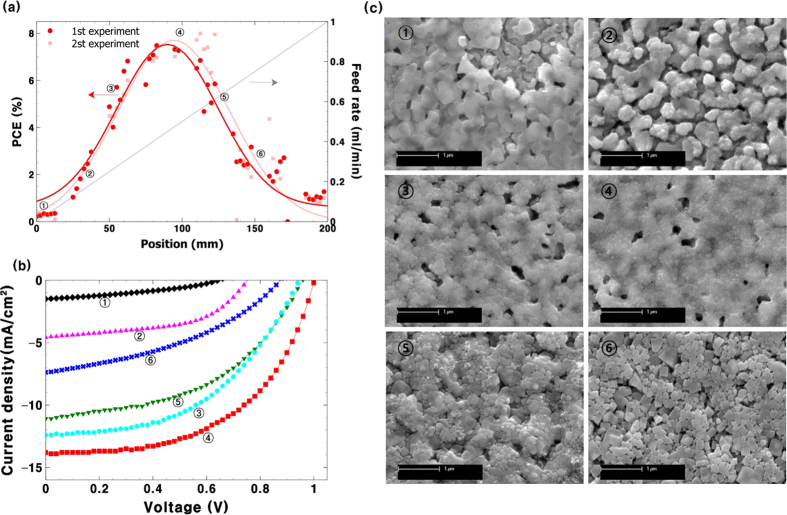
(**a**) PCE (**b**) J-V curve of perovskite solar cells with spray deposited MAI with gradually increased solution flow rates on slot die coated PbI_2_ layers (**c**) SEM images of the spray deposited perovskite layers at different flow regimes.

The sequential deposition process consists of PbI_2_ layer coating and a conversion step typically performed by a dipping process. To prepare PbI_2_ layer, we tried spray deposition first and found that forming defect free uniform PbI_2_ layer by spray coating was very challenging due to rapid crystallization of PbI_2_. We already experienced same problem in slot die coating process and found a solution for the issue. Therefore, uniform PbI_2_ layers were prepared by slot die coating as we previously reported^9^. Eight samples were placed under the spray coater and a solution of methylammonium iodide (MAI) in 2-propanol was deposited by differential solution pumping as used in the PSCs. In this case, the single-feed mode was used because there is only one condition to be varied in this experiment. Solution flow was increased from 0.01 mL/min to 1 mL/min while keeping the carrier gas flow and the coating speed constant. To stop the overflow of solution and to accelerate the conversion reaction, substrates were heated to 90 °C. The films immediately changed from yellow to brown with the MAI deposition. The deposition of P3HT as a hole transporting layer and a silver electrode completed the devices. Figure 4(a) shows the PCE trend of PeSCs depending on the flow rate of the MAI solution. At the starting point, with the flow rate <0.1 ml/min, devices showed ~0% PCE and the PCE sharply increased with an increase in flow rate. This finding was expected as the conversion requires more than 1 equivalent of MAI and 0.01 mL/min was expected to have much less than that. With increased solution flow, the degree of conversion increased. This could be checked by conducting a thickness measurement after PbI_2_ was converted. We found that the films reached ~500 nm in thickness after full conversion by the dipping process. The spray deposited films showed gradually increased thickness and reached a saturated thickness (~500 nm) at ~0.4 ml/min region. The best device performance was also obtained near the maximum flow rate (~0.4 ml/min). The current density-voltage (J-V) characteristics of the devices are shown in Fig. 4(b) and a summary of device parameters can be seen in Table 1. The device at the ① region showed a poor open circuit voltage (V_oc_), a poor short circuit current density (J_sc_) and a poor fill factor (FF). All device parameters were increased and reached maximum at ④ (~0.50 ml/min flow rate) region. The best device at the first experiment showed V_oc_ = 1.00 V, J_sc_ = 13.81 mA/cm^2^, FF = 54.15 and PCE = 7.48% and that of second experiment showed V_oc _= 1.00 V, J_sc _= 15.08 mA/cm^2^, FF = 52.99 and PCE = 7.99%. The efficiency of devices was sharply decreased once past the optimum point in repeated experiments.

The reason for decreased efficiency can be attributed to excess amount of MAI that can form insulating layer on top of perovskite layer. However, our previous experience has shown that an excess amount of MAI is not critical to the performance of devices in the conversion of PbI_2_ to perovskite by either dipping or slot die coating as excess amounts of MAI could be easily washed away with 2-propanol. Therefore, we analysed the films using a scanning electron microscope (SEM) to find the origin of the performance decay. Actual devices were used to obtain SEM images instead of preparing separate samples to avoid batch-to-batch variation. The P3HT layer could be easily washed away by means of ultrasonication in a chlorobenzene bath within ten seconds. Metal electrodes on top of the polymer layer were removed. SEM images of the perovskite layers of the devices are shown in Fig. 4(c). Unreacted PbI_2_ film is seen in the ① region. The image looks similar to that of a PbI_2_ film before the soaking process^9^. The films start to grow from the ② region and form a well-connected crystalline perovskite phase in the ③ and ④ regions. However, the films were not defect free so the performance in general was lower than is typical in sequentially processed devices. The source of the performance decay in the ⑤ and ⑥ region could be deduced from the SEM images. It appeared that with increased solution flow, a larger number of discrete crystal domains were formed and the size of each domain was much smaller than the film thickness (~500 nm). It is well known that a discrete grain boundary is critical for charge transportation^41^. Therefore, charges were likely trapped and/or recombined given the large number of crystal domains observed.

From our experiments, various deposition conditions could be tested and an optimum deposition condition at a given concentration and temperature could be easily found. While further process optimisation is always possible, our results clearly show the potential of spray coating as a screening process for both PSCs and PeSCs. New materials can also be efficiently tested using a wide range of deposition conditions to quickly establish their potential.

## Conclusion

We demonstrated that a spray coating system can be used as a material screening tool for PSCs and PeSCs. By having automated position control and a dual-feed nozzle, in the case of PSCs, various deposition conditions including the blend ratio of the donor/acceptor materials and the solution flow rates could be screened in a single experiment. Polymer solar cells based on P3HT were fabricated by a dual-feed spray approach with various donor/acceptor ratios in a single deposition process and a PCE of up to 3% was found. Although the efficiencies measured were not as good as typically found in optimised devices, it was determined that the optimum blend ratio obtained from a single experiment is consistent with the values reported in the literature. Therefore, we believe that this rapid screening method will be useful for finding good starting points for further optimization. In addition to having an application in PSCs, the method was used to find the optimum deposition parameters for the fabrication of PeSCs via a sequential deposition process. It was found that the deposition rate of MAI has a significant impact on the formation of perovskite films. Distinctive forms of perovskite layers were found in different deposition regimes and the optimum deposition parameter could be found from a single experiment. The best spray deposited cell demonstrated up to 7.99% PCE. We believe the reported method can be widely used for various solution processed solar cells and will be especially useful for the optimization of new materials.

## Methods

### Device fabrication

ITO coated glass (Shenzhen Display, 5Ω/□) was cleaned with Deconex 12PA detergent solution, deionised water, acetone and isopropanol in an ultrasonic bath for 5 min successively and exposed to UV-ozone for 15 min. Poly(3,4-ethylenedioxythiophene)-poly(styrenesulfonate) (PEDOT:PSS) was dispensed through a syringe filter (0.2 μm RC filter) on the cleaned ITO substrates and spin coated at 5000 rpm for 20 sec. The films were then baked at 140 °C for 10 min in air and transferred to an N_2 _filled glove box for spray deposition. For the P3HT:PC_61_BM based PSCs, 5 mg of P3HT (Merck) and 5 mg of PC_61_BM (Nano-C) were dissolved in 1 mL of chlorobenzene separately. Each solution was transferred to a syringe and deposited by a spray process on PEDOT:PSS. Eight PEDOT:PSS coated substrates (25 mm × 25 mm) were placed below the spray nozzle. The solution feed ratio was controlled by syringe pumps from 0.01 to 0.07 ml/min for P3HT and from 0.077 to 0.011 ml/min for PC_61_BM while moving from one end of the substrate to the other, for all eight samples. The thickness of each deposition layer was controlled by total deposition time while maintaining a constant total solution flow rate. 100 nm of Al was evaporated at ~10^−7^ torr pressure. Before measuring the J-V curve, the devices were annealed at 140 °C for 10 min.

For PeSCs, ZnO/PbI_2_ films were prepared on cleaned ITO glass in air as previously reported^40^. The films were transferred to an N_2_ filled glove box for spray deposition of MAI. Eight substrates were placed on a hot plate below the spray nozzle. To increase reactivity with MAI, PbI_2_ films were heated by the hot plate at 90 °C. The substrates were pre-heated for 5 min and then the MAI solution was deposited at an increasing solution flow rate ranging from 0.01 to 1 mL/min while moving from one end of the substrate to the other for 350 sec. After the deposition process, the films were washed with 2-propanol to remove unreacted MAI on the surface. For the HTL fabrication, 15 mg of P3HT was dissolved in 1 ml of chlorobenzene (CB) and 6.8 μL Li-bis (trifluoromethanesulfonyl) imide (Li-TFSI) in acetonitrile (28.3 mg/ml) and 3.4 μL 4-tert-Butylpyridine (TBP) were added to the solution. The solution was spin-coated on the perovskite films at 600 rpm for 12 sec and then 2000 rpm for 40 sec in air. The films were transferred and 100 nm of Ag was evaporated at ~10^−7^ torr pressure.

### Measurement

The thickness of deposited films was measured using a Dektak 6M surface profiler. To observe the surface of the perovskite film, secondary electron microscope (SEM, Philips XL30 FEG) was measured. To prepare SEM samples, the devices with measured J-V characteristics were washed using chlorobenzene solvent in an ultrasonic bath to remove P3HT and metal electrode layers. The J-V characteristics of the small devices were measured in an inert atmosphere with a computer-controlled Keithley 2400 Source Measure Unit(SMU). A 150 W Xenon lamp (Newport) coupled with an AM 1.5G solar spectrum filter was used as the light source. Light was introduced through a quartz window in the glove box and the intensity was calibrated and monitored using a secondary reference cell (Hamamatsu S1133, with KG-5 filter, 2.8 × 2.4 mm of photosensitive area) which was calibrated by a certified reference cell (PV Measurements, certified by NREL) under 1000 W/m^2^ AM 1.5 G illumination from an Oriel AAA solar simulator fitted with a 1000 W Xe lamp. Light intensity of the secondary reference cell and a sample with 6 cells were mounted on a motorized stage. Light input was monitored by the secondary reference cell at all time except when samples were measured. To measure each cell, the stage moved to position measuring cell at the calibrated spot by the reference cell and PC controlled electrical switch connected the cell to SMU. Therefore, all cells were measured at the same physical position to eliminate error due to light intensity issue. The calibrated intensity was also periodically re-confirmed using encapsulated OSCs with same device design under an AAA solar simulator.

## Additional Information

**How to cite this article**: Jung, Y.-S. *et al.* Differentially pumped spray deposition as a rapid screening tool for organic and perovskite solar cells. *Sci. Rep.* **6**, 20357; doi: 10.1038/srep20357 (2016).

## Figures and Tables

**Table 1:** Summary of device characteristics for perovskite based solar cells based on variations in flow rate of MAI.

	**Flow rate**	**V**_**OC**_ **(V)**	**J**_**SC**_**(mA/cm**^2^)	**FF (%)**	**PCE (%)**
①	0.05	0.64	1.50	35.6	0.34
③	0.20	0.76	4.58	52.4	1.82
④	0.30	0.94	12.39	49.0	5.71
⑤	0.45	1.00	13.81	54.1	7.48
⑥	0.65	0.96	11.09	47.4	5.05
⑦	0.85	0.88	7.38	39.3	2.55
